# Supplementary figures and images for: The Antiviral Effect of Isatis Root Polysaccharide against NADC30-like PRRSV by Transcriptome and Proteome Analysis
Source: Int J Mol Sci. 2022 Mar 28;23(7):3688. doi: 10.3390/ijms23073688 (PMC8998840; doi:10.3390/ijms23073688)

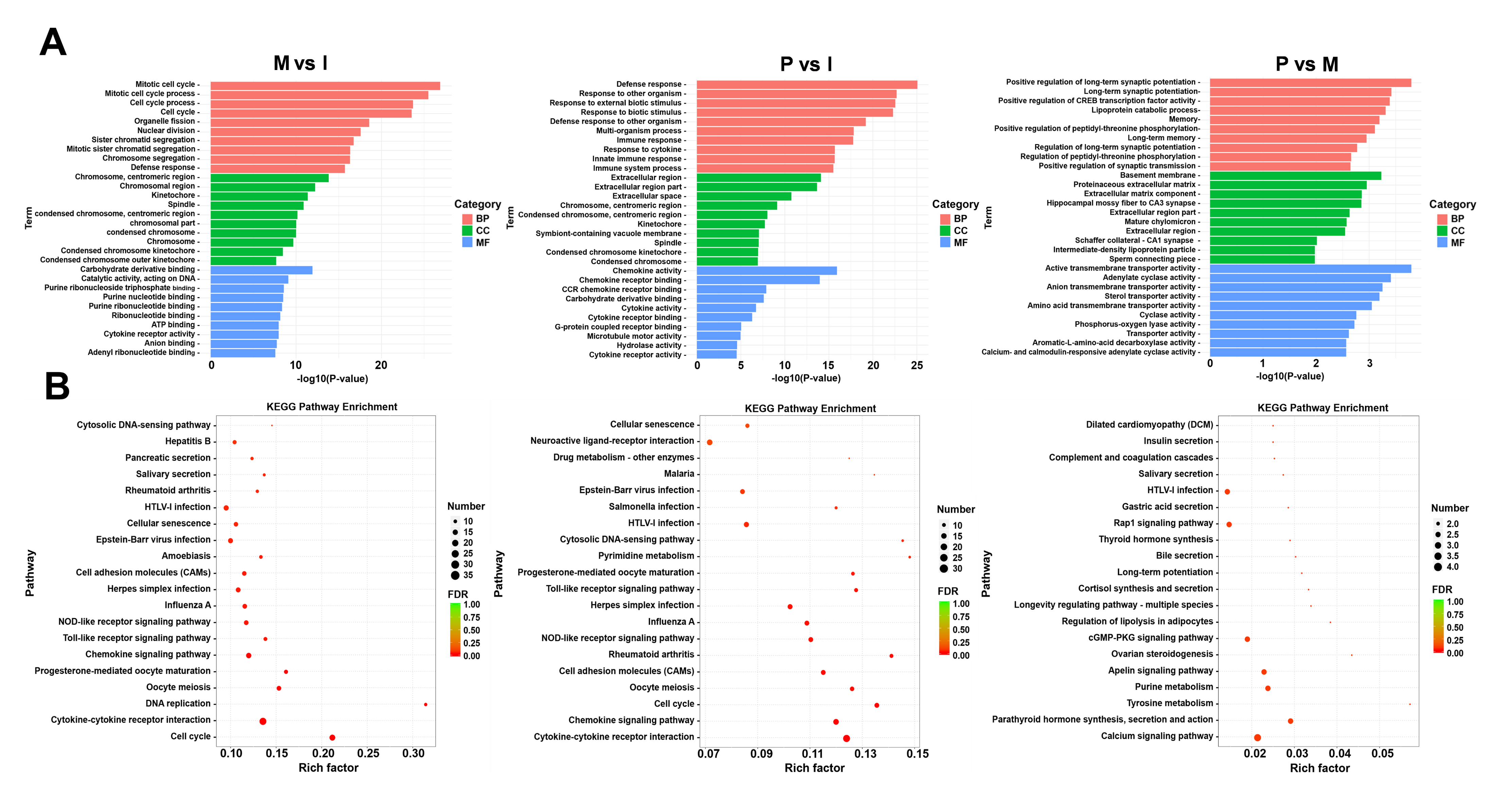

Supplement: Supplementary file 1 [file ijms-23-03688-s001.zip › Figure S1.tif]

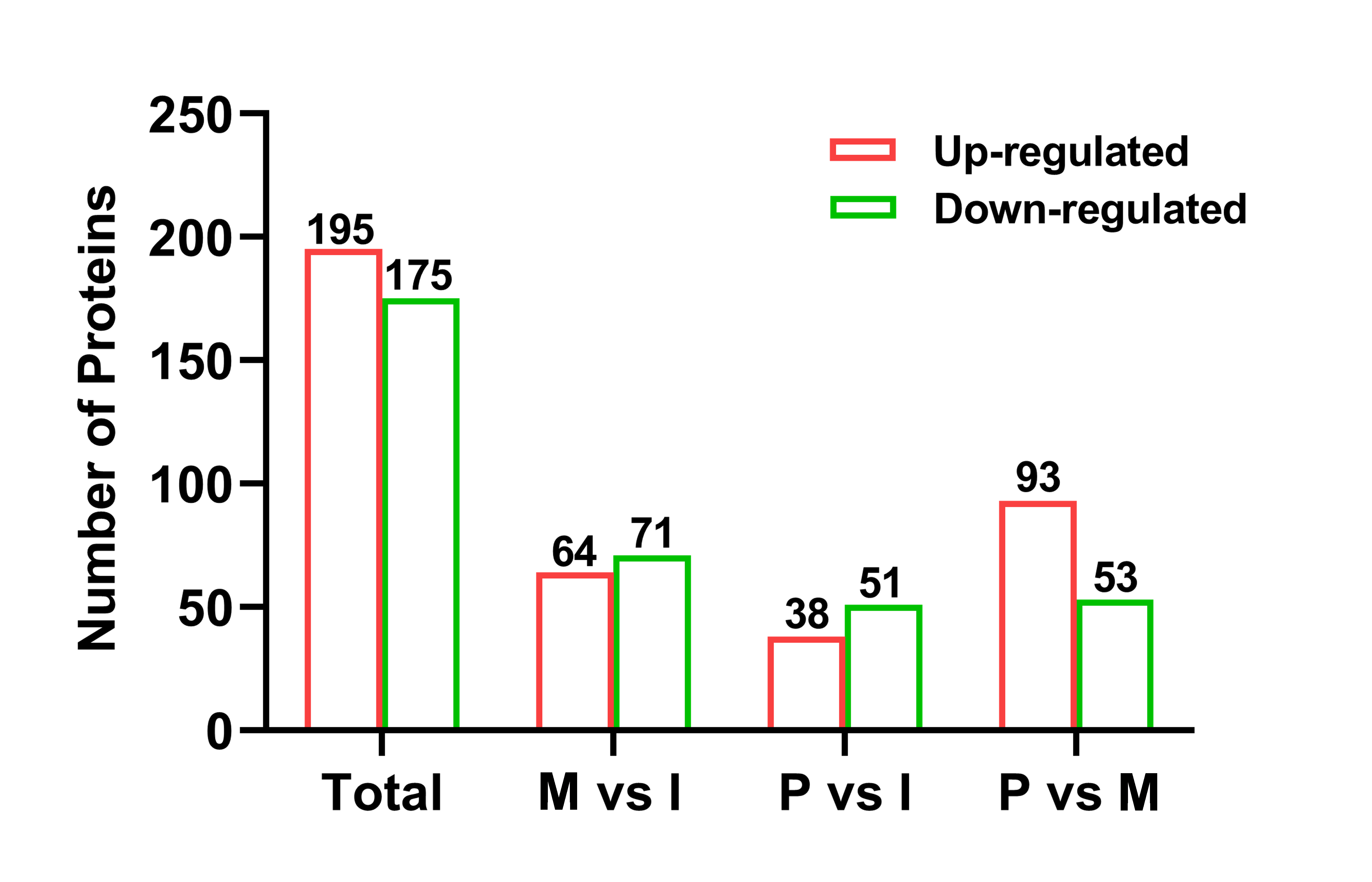

Supplement: Supplementary file 1 [file ijms-23-03688-s001.zip › Figure S2.tif]

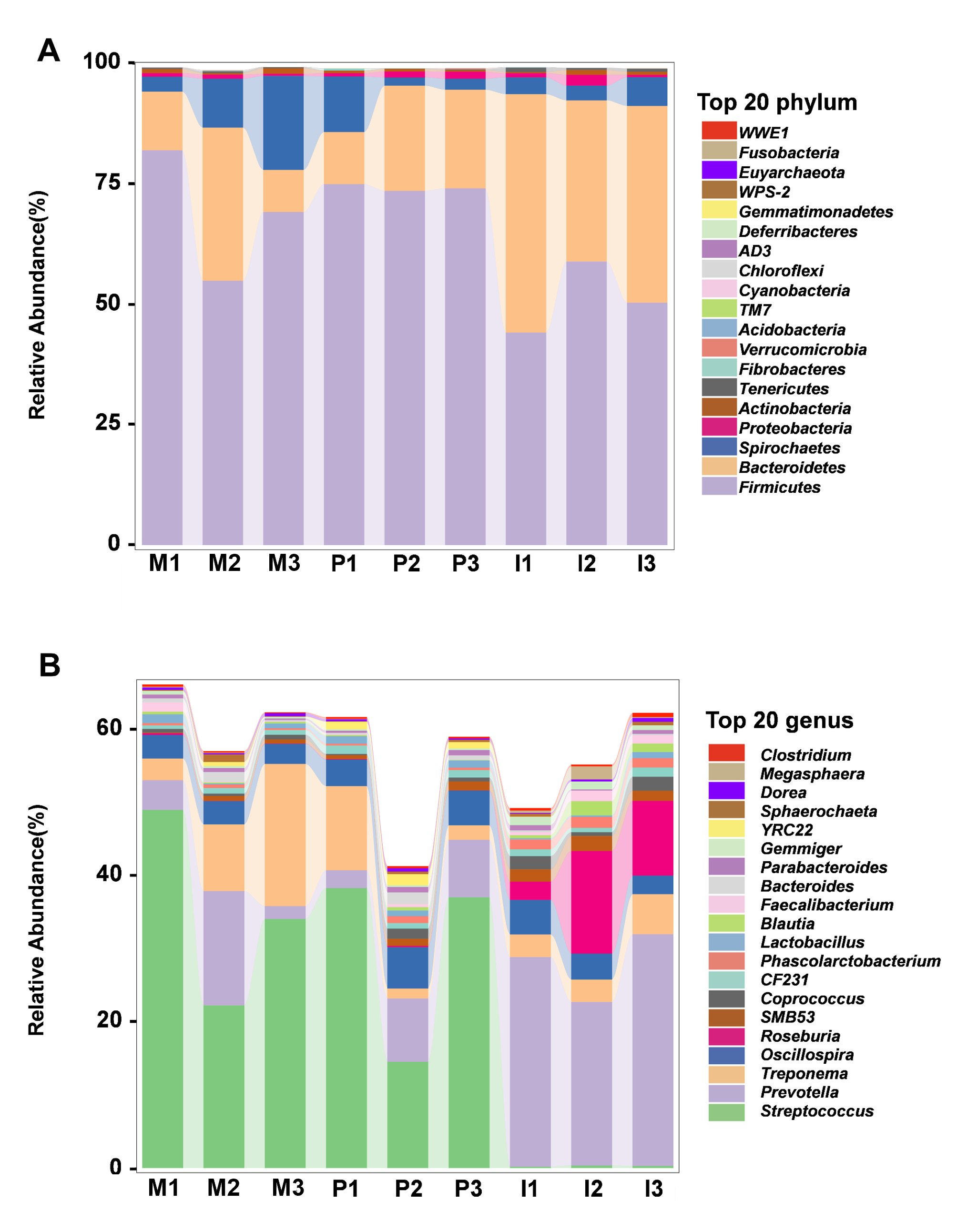

Supplement: Supplementary file 1 [file ijms-23-03688-s001.zip › Figure S3.tif]
